# Deep learning-based thigh muscle segmentation for reproducible fat fraction quantification using fat–water decomposition MRI

**DOI:** 10.1186/s13244-020-00946-8

**Published:** 2020-11-30

**Authors:** Jie Ding, Peng Cao, Hing-Chiu Chang, Yuan Gao, Sophelia Hoi Shan Chan, Varut Vardhanabhuti

**Affiliations:** 1 Department of Diagnostic Radiology, Li Ka Shing Faculty of Medicine, The University of Hong Kong, Pok Fu Lam, Hong Kong SAR China; 2 Department of Radiation Oncology, Medical College of Wisconsin, Milwaukee, WI 53226 USA; 3 Division of Neurology, Department of Medicine, Queen Mary Hospital, The University of Hong Kong, Pok Fu Lam, Hong Kong SAR China; 4 Division of Paediatric Neurology, Department of Paediatrics and Adolescent Medicine, Li Ka Shing Faculty of Medicine, The University of Hong Kong, Pok Fu Lam, Hong Kong SAR China

**Keywords:** Thigh muscle segmentation, Deep learning, Fat–water decomposition MRI, Quantitative MRI analysis

## Abstract

**Background:**

Time-efficient and accurate whole volume thigh muscle segmentation is a major challenge in moving from qualitative assessment of thigh muscle MRI to more quantitative methods. This study developed an automated whole thigh muscle segmentation method using deep learning for reproducible fat fraction quantification on fat–water decomposition MRI.

**Results:**

This study was performed using a public reference database (Dataset 1, 25 scans) and a local clinical dataset (Dataset 2, 21 scans). A U-net was trained using 23 scans (16 from Dataset 1, seven from Dataset 2) to automatically segment four functional muscle groups: quadriceps femoris, sartorius, gracilis and hamstring. The segmentation accuracy was evaluated on an independent testing set (3 × 3 repeated scans in Dataset 1 and four scans in Dataset 2). The average Dice coefficients between manual and automated segmentation were > 0.85. The average percent difference (absolute) in volume was 7.57%, and the average difference (absolute) in mean fat fraction (meanFF) was 0.17%. The reproducibility in meanFF was calculated using intraclass correlation coefficients (ICCs) for the repeated scans, and automated segmentation produced overall higher ICCs than manual segmentation (0.921 vs. 0.902). A preliminary quantitative analysis was performed using two-sample *t* test to detect possible differences in meanFF between 14 normal and 14 abnormal (with fat infiltration) thighs in Dataset 2 using automated segmentation, and significantly higher meanFF was detected in abnormal thighs.

**Conclusions:**

This automated thigh muscle segmentation exhibits excellent accuracy and higher reproducibility in fat fraction estimation compared to manual segmentation, which can be further used for quantifying fat infiltration in thigh muscles.

## Key points

- This fully automated deep learning-based thigh muscle segmentation exhibits excellent accuracy.
- It can delineate four clinically relevant thigh muscle groups in seconds.
- It provides higher reproducibility in fat fraction estimations compared to manual segmentation.

## Background

Previous studies have shown that fat infiltration can be observed in thigh muscles due to diseases such as neuromuscular and metabolic disorders or age-related muscle atrophy (sarcopenia) [1–4]. More importantly, the degree of intramuscular fat infiltration serves as a marker for disease severity and progression [5–10].

Quantitative magnetic resonance imaging (MRI) provides an excellent approach for noninvasively measuring the fat composition in various organs, including muscle tissue. Fat–water decomposition MRI, one of the quantitative MRI techniques, is highly suitable for fat fraction quantification due to its ability to separate MRI signals from water and fat protons based on their chemical shift difference. This technique was first proposed by Dixon [11] as the original two-point Dixon method, which acquires two sets of images at echo times when water and fat protons are in phase and out of phase [11]. With the advancement of MRI techniques, multipoint Dixon methods [12, 13] have been developed to acquire images at three or more echo times with different phase shifts between fat and water protons for fat–water separation. Compared to two-point Dixon method, three-point Dixon sequences [12] are able to correct the magnetic field inhomogeneities, which offer separated fat and water images for quantitative analysis. Some advanced multipoint Dixon techniques can address other confounding factors and provide more accurate fat and water images and the corresponding proton density fat fraction (PDFF) maps [14, 15]. Currently, multipoint Dixon sequences have been clinically implemented and also widely used in research studies for quantifying intramuscular fat infiltration in the thigh [2–9, 15–20].

However, the need for segmentation severely limits the application of fat–water decomposition MRI in quantitative fat assessment in clinical practice. Image segmentation is the first and crucial step in order to quantitatively evaluate the intramuscular fat fraction in the thigh. The conventional methods for segmenting thigh muscles in the current literature [3–5, 7–9, 15–20] rely on manually drawn regions-of-interest (ROIs), which is not only an extremely time-consuming and cumbersome process but also prone to subjective bias and inter-reader variation. A previous study [20] showed the average time for segmenting four entire muscle regions was approximately 6 h for each scan. Hence, most previous studies performed manual segmentation on one or a few representative slices [3–5, 7–9, 15–17]. Such a process can lower the accuracy and reproducibility, which is also not feasible for clinical adoption. Although recent studies developed semiautomated or automated algorithms [21–25], they are either based on nonquantitative MRI or difficult to apply in clinical settings [26]. A faster, simpler and fully automated thigh muscle segmentation method for reproducible fat fraction quantification is highly desired.

In recent years, deep learning techniques, in particular convolutional neural networks (CNNs), have been successfully applied in medical image analysis field, notably in image segmentation [27]. CNNs [28, 29] are trainable models established by multiple layers with operations including convolution, pooling and activation, which can capture the highly nonlinear mappings between inputs and outputs. This approach provides a powerful tool for image segmentation, as CNNs can automatically learn and leverage the representative patterns of the training images and then make accurate predictions on prospective images after optimization. U-net [30], one of the most well-known CNN architectures, is designed particularly for semantic segmentation of biomedical images. This network utilizes an encoder–decoder structure with skip connections and is able to extract higher resolution features more efficiently. U-net has been applied in plenty of studies for medical image segmentation and achieved promising and robust results [27]. However, the performance of such technique on thigh muscle segmentation has not yet been investigated and evaluated in the current literature.

The purpose of this study was to train and validate a CNN with sufficient accuracy for automatically segmenting four functional thigh muscle groups using fat–water decomposition MRI. We further evaluated its reproducibility in fat fraction estimation by comparing with manual segmentation and performed a preliminary quantitative analysis showing its ability in differentiating the abnormal thighs with fat infiltration.

## Materials and methods

This study was approved by the institutional review board. Owing to the fact that this is a retrospective study, informed consent was waived.

### Datasets

**Fig. 1:**
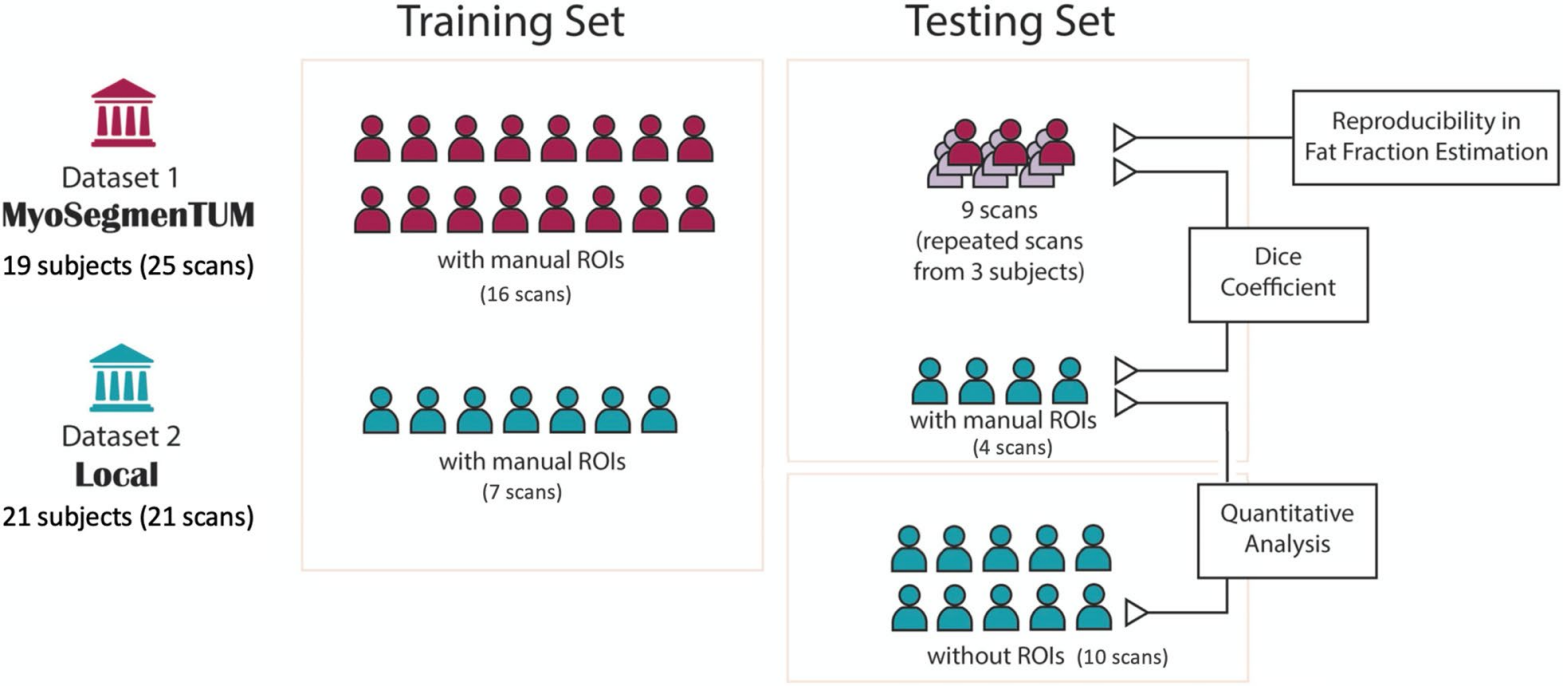
An overview of the two datasets and workflow of this study

Figure 1 provides an overview of the datasets used in this study. Detailed demographic and clinical characteristics are provided in Additional file 1: Table S1.

#### Dataset 1: a public reference database MyoSegmenTUM [20]

This database consists of 25 fat–water decomposition MRI scans collected from 19 subjects (15 healthy volunteers and 4 patients with neuromuscular diseases) using 6-echo 3D spoiled gradient echo (GRE) sequences. Details on MRI acquisitions were described in the reference [20]. Three healthy subjects were scanned three times with repositioning for reproducibility assessment. The manual segmentation masks of all scans were provided in the database as ground truth. The manual ROIs delineated four clinically relevant muscle groups (whole volume in 3D): quadriceps femoris muscle (ROI1), sartorius muscle (ROI2), gracilis muscle (ROI3) and hamstring muscles (ROI4), with an average segmentation time of ~ 6 h per scan.

#### Dataset 2: local clinical data

Fat–water decomposition MRI images of 21 subjects were collected between 2018 and 2020 for clinical purpose in our site on a 1.5-T GE Signa scanner, using a 3D three-point Dixon method and an iterative algorithm IDEAL [13] for fat–water decomposition. Images were acquired with the following parameters: repetition time (TR) = 7.608 ms, effective echo time (TE) = 3.576 ms, flip angle = 8°, reconstructed matrix size = 512 × 512,voxel size = 0.7422 × 0.7422 × 6mm^3^, the band width of 244 Hz/voxel. Scanning was performed with two consecutive stacks (upper and lower portions of the thigh) in the axial plane to cover the whole bilateral thigh volumes using GE HD 12-channel body array coil, and each stack had approximately 40 slices. The manual segmentation masks of 11 scans were drawn by an operator and examined and approved by a radiologist with 12 years’ experience. The manual masks contained the same four ROIs as Dataset 1.

### MRI preprocessing

For all scans, the left and right sides of the thighs were first separated and treated as two thigh volumes for analysis, which also doubled the dataset size. As a preprocessing step, the subcutaneous fat and skin were removed automatically on water images to potentially improve the segmentation performance: (1) A K-means clustering was first performed on water images and the cluster with higher intensity was kept (subcutaneous fat with lower intensity was removed); (2) the skin region was then removed by applying an order-statistic filtering on the mask generated in the last step; and (3) the final mask was generated after filling the holes and a dilation (in case muscle regions were incorrectly removed in the previous steps). The MRI preprocessing was performed in MATLAB R2019a. The water and fat images covering only the muscle regions for each thigh volume were used for the CNN training and evaluation.

### Convolutional neural network training

**Fig. 2:**
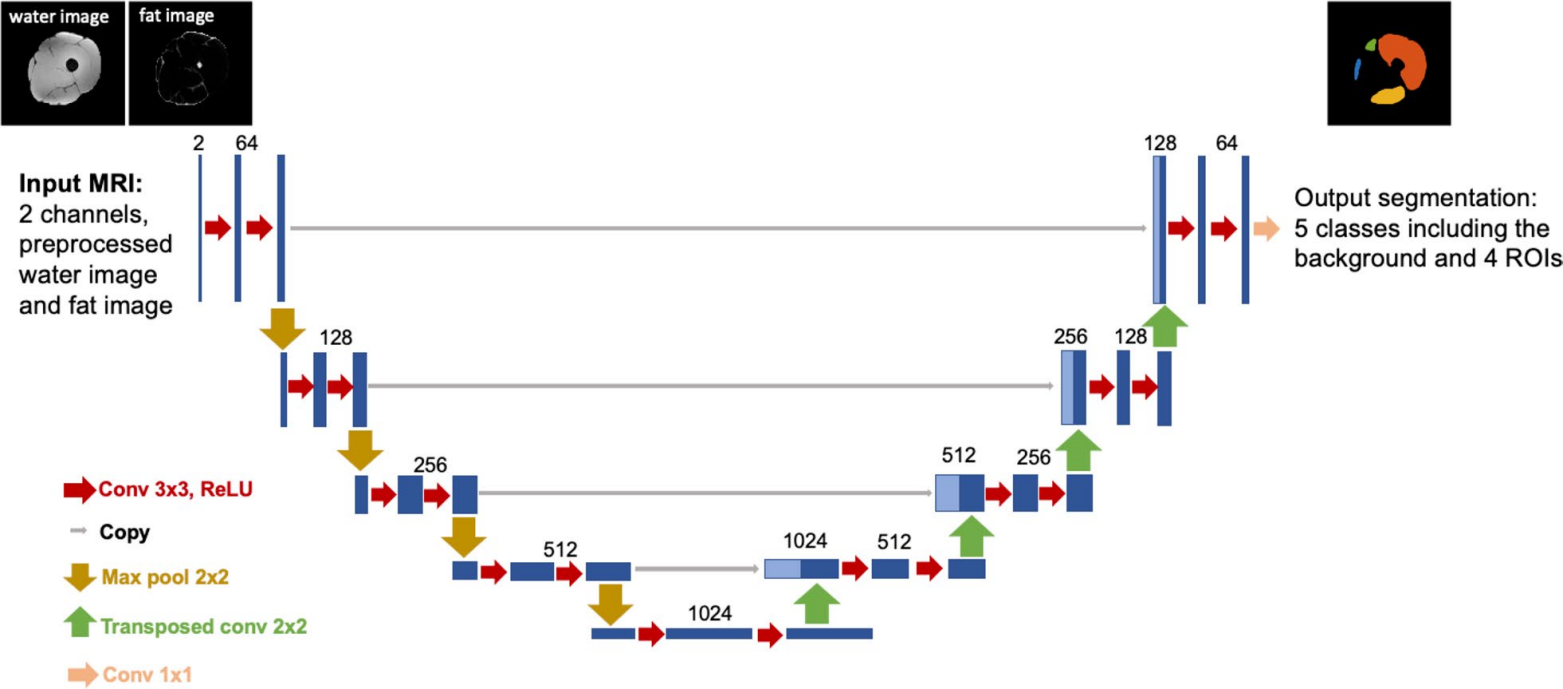
U-net architecture for segmentation of the four thigh muscle regions

A CNN was trained to automatically segment the four thigh muscle groups (ROI1–ROI4) using a U-net architecture [30] in Keras [31] with TensorFlow backend [32] on a Windows workstation with an Nvidia 1080Ti GPU. The network architecture is shown in Fig. 2. The whole dataset with manual ROIs for developing the CNN consisted of 25 scans from Dataset 1 and 11 scans from Dataset 2. The network was trained in 2D using a total of 4968 slices from 46 thigh volumes (23 scans, 16 from Dataset 1 and seven from Dataset 2). The preprocessed water and fat images were used as two input channels, and both channels were independently normalized to [0, 1]. The manual segmentation masks were provided as ground truth. The network was trained using a batch size of 6 for 1000 epochs on ~ 3/4 of the training set (3940 slices from 36 thigh volumes of 18 scans, 13 from Dataset 1 and five from Dataset 2). Validation was performed after each epoch using on the rest ~ 1/4 of the training set (1028 slices from ten thigh volumes of five scans, three from Dataset 1 and two from Dataset 2). The network was trained using Adam optimizer with a learning rate of 10^−6^ [33]. The training objective was to minimize the categorical cross-entropy loss between the CNN outputs and the manual segmentations. The CNN outputs were compressed to the range (0,1) using a sigmoid function. A threshold of 0.5 was applied to binarize the CNN outputs. The binarized CNN outputs were stacked back to thigh volumes, and for each ROI, only the largest region in 3D was kept as the final automated mask.

### Convolutional neural network evaluation

The CNN segmentation was evaluated using an independent testing set consisting of those MRI scans that were not used for CNN training but had manual ROIs. The testing set included the MRI images acquired from the three subjects with three repeated scans in Dataset 1 (3 × 3 scans, 6 × 3 thigh volumes) and four scans in Dataset 2 (eight thigh volumes). The performance of the CNN segmentation was assessed by comparing with manual segmentation in three ways: (1) Dice coefficient at the volume level; (2) differences in volume and fat fraction estimation; the percent differences in volume between manual and CNN segmentation were assessed; for each scan, a fat fraction map was generated as the ratio of fat image over the sum of water and fat images, and the mean fat fraction (meanFF) values in each muscle group were calculated. (3) Reproducibility in meanFF determined by the intraclass correlation coefficients (ICCs) in each ROI using the repeated scans in Dataset 1. For (1) and (2), in order to avoid bias from the repeated scans, the values of each thigh volume with repeated scans were averaged for assessment.

### A quantitative analysis using CNN segmentation

A preliminary quantitative analysis was performed using the clinical scans of 14 subjects in Dataset 2 that were not used for CNN training. As few patients received biopsy-based pathological examination, radiological assessment was used as a reference standard. Among the 14 subjects, seven of them had no radiological evidence of abnormal fat infiltration in their thigh muscles, while the remaining seven subjects showed abnormal fat replacement of muscle (14 normal vs. 14 abnormal thigh volumes). The meanFF values in each ROI were calculated using the CNN segmentation masks. A one-tailed, two-sample *t* test assuming unequal variances was performed to detect possible differences in meanFF between normal and abnormal thighs. A *p* value of < 0.05 considered statistically significant.

## Results

### Performance of the CNN segmentation

**Fig. 3:**
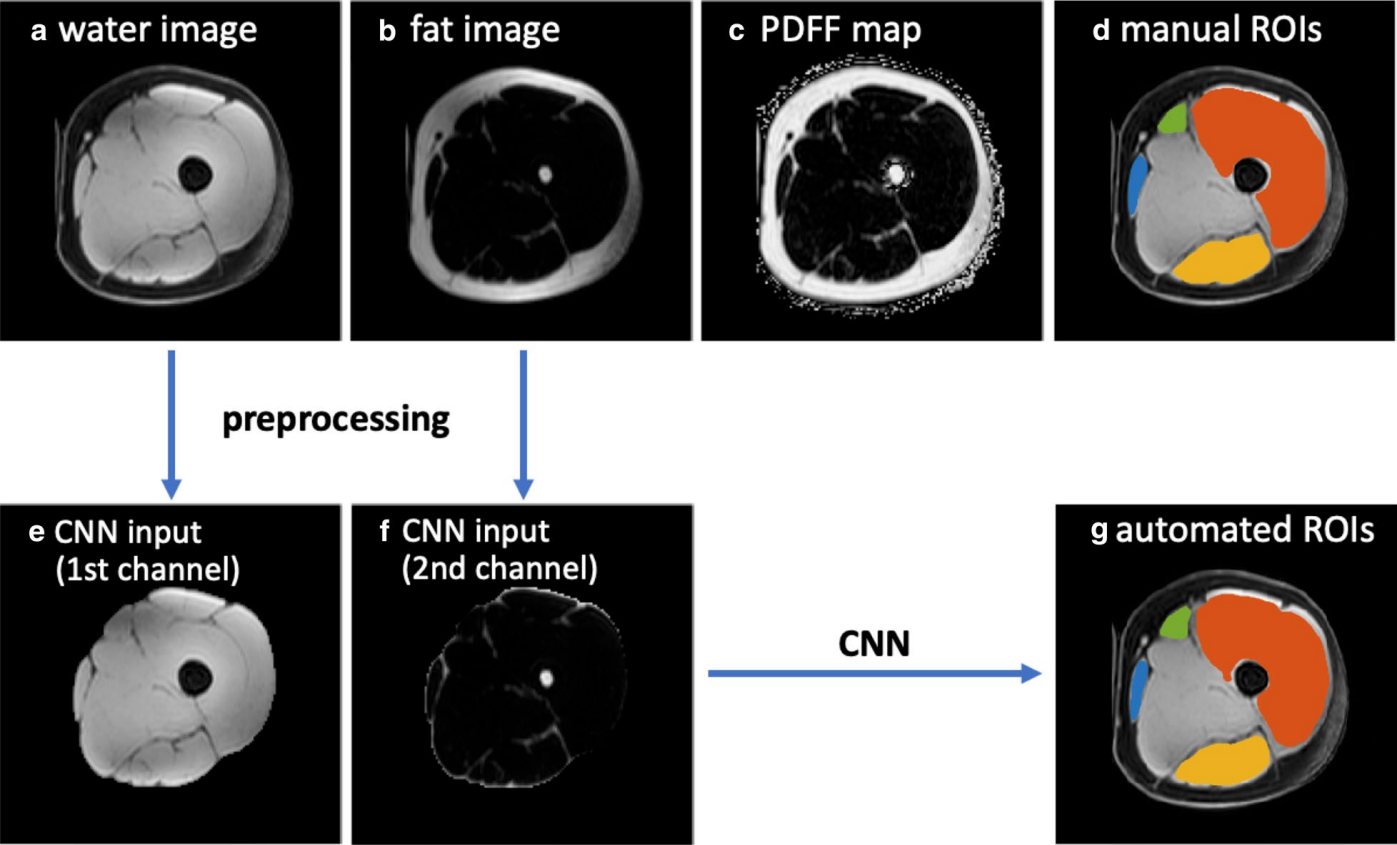
A scheme of this study, with MRI images and the segmentation results of an example from Dataset 1 in the testing set. **a**–**c** the original water image, fat image and PDFF map provided by Dataset 1, **d** the manual segmentation masks of four ROIs, **e**, **f** the preprocessed water image and fat image covering the whole thigh muscles as CNN input channels, **g** the automated CNN-derived segmentation masks. For **d**, **g**, segmentations are superimposed on the water image, with ROI1 (quadriceps femoris) in red, ROI2 (sartorius) in green, ROI3 (gracilis) in blue and ROI4 (hamstrings) in yellow

**Fig. 4:**
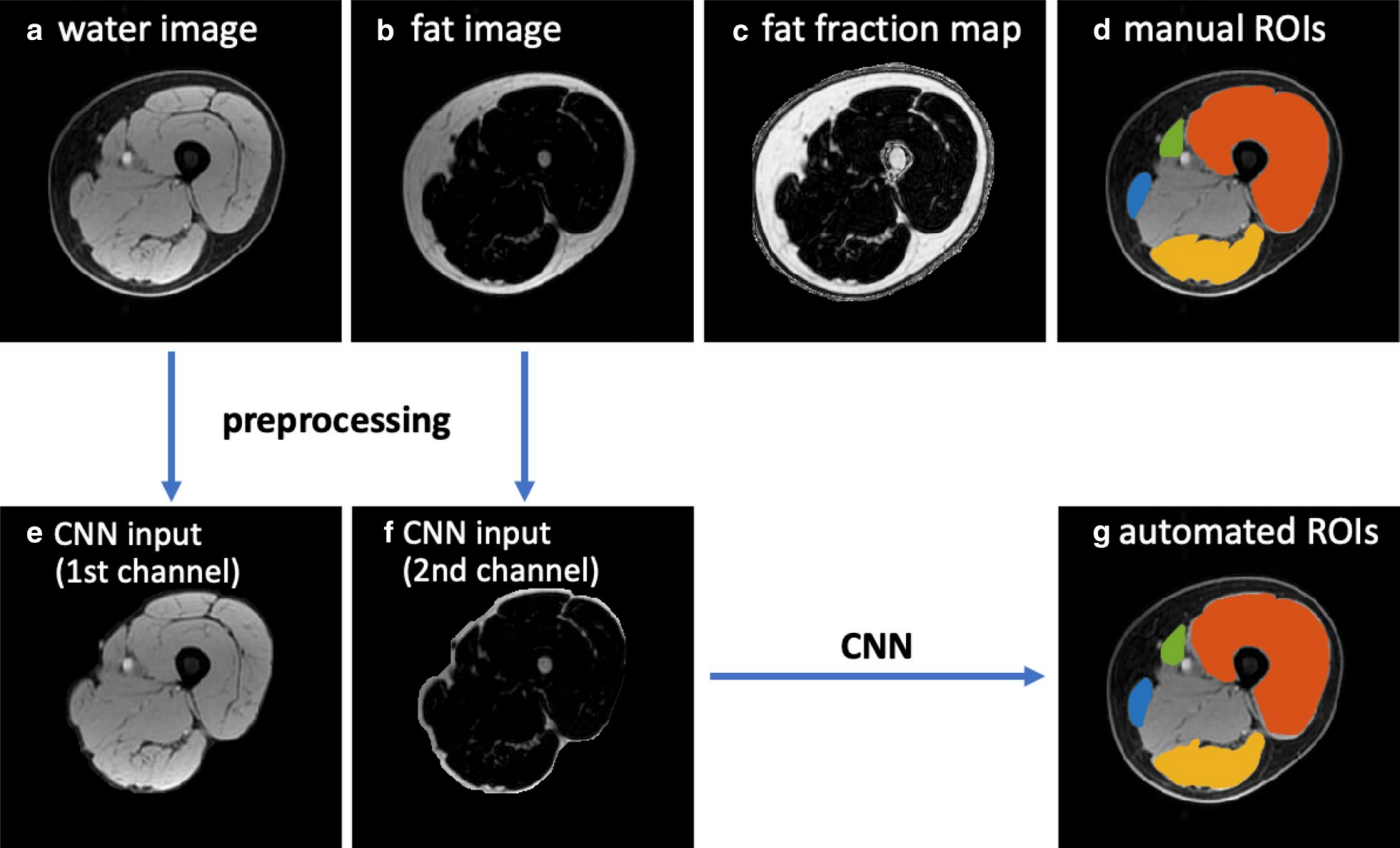
MRI images and the segmentation results of an example from Dataset 2 in the testing set. **a**–**c** the original water image, fat image and the calculated fat fraction map, **d** the manual segmentation masks of four ROIs, **e**, **f** the preprocessed water image and fat image covering the whole thigh muscles as CNN input channels, **g** the automated CNN-derived segmentation masks. For **d**, **g**, segmentations are superimposed on the water image, with ROI1 (quadriceps femoris) in red, ROI2 (sartorius) in green, ROI3 (gracilis) in blue and ROI4 (hamstrings) in yellow

Figures 3 and 4 show the MRI images and the segmentation results of a representative slice in the independent testing set from Dataset 1 and Dataset 2, respectively. The manual segmentation and automated segmentation show excellent agreement visually. The CNN generated the ROIs within 10–30 s for each thigh volume.

Table 1 summarizes the mean and standard deviation Dice coefficients between manual and automated segmentation results for ROI1 ~ ROI4 of each thigh volume in the testing set. To avoid bias, the Dice coefficients of each thigh volume with repeated scans were first averaged (their mean ± standard deviation was calculated as shown in Table 1). It is noted that some ROIs are very small, or some regions (particularly at either end of muscle insertion/attachment) can be more inaccurate, which impacts the Dice coefficient. Our mean Dice coefficients were > 0.85 for all ROIs, even for the smallest ROI2. The Dice coefficients of ROI2 and ROI3 were relatively lower than those of ROI1 and ROI4 because ROI2 and ROI3 are smaller muscle groups.

**Table 1:** Segmentation accuracy assessed by Dice coefficients between manual and automated segmentation for each ROI of each thigh volume in the testing set

	ROI1	ROI2	ROI3	ROI4
Dataset 1 (mean ± SD as repeated scans were performed)				
1st, left	0.942 ± 0.002	0.886 ± 0.007	0.866 ± 0.015	0.915 ± 0.004
1st, right	0.923 ± 0.010	0.812 ± 0.038	0.866 ± 0.011	0.900 ± 0.014
2nd, left	0.927 ± 0.006	0.848 ± 0.005	0.846 ± 0.024	0.917 ± 0.004
2nd, right	0.927 ± 0.013	0.872 ± 0.007	0.877 ± 0.013	0.902 ± 0.024
3rd, left	0.933 ± 0.003	0.869 ± 0.007	0.861 ± 0.003	0.925 ± 0.003
3rd, right	0.932 ± 0.011	0.874 ± 0.004	0.876 ± 0.021	0.915 ± 0.014
Dataset 2				
1st, left	0.958	0.890	0.881	0.905
1st, right	0.954	0.918	0.897	0.911
2nd, left	0.923	0.848	0.819	0.842
2nd, right	0.920	0.774	0.835	0.824
3rd, left	0.944	0.806	0.819	0.904
3rd, right	0.946	0.814	0.771	0.871
4th, left	0.960	0.914	0.931	0.897
4th, right	0.959	0.904	0.902	0.884
Mean	0.939	0.859	0.860	0.894
SD	0.015	0.044	0.040	0.029

ROI1, quadriceps femoris; ROI2, sartorius; ROI3, gracilis; ROI4, hamstrings; SD, standard deviation

**Fig. 5:**
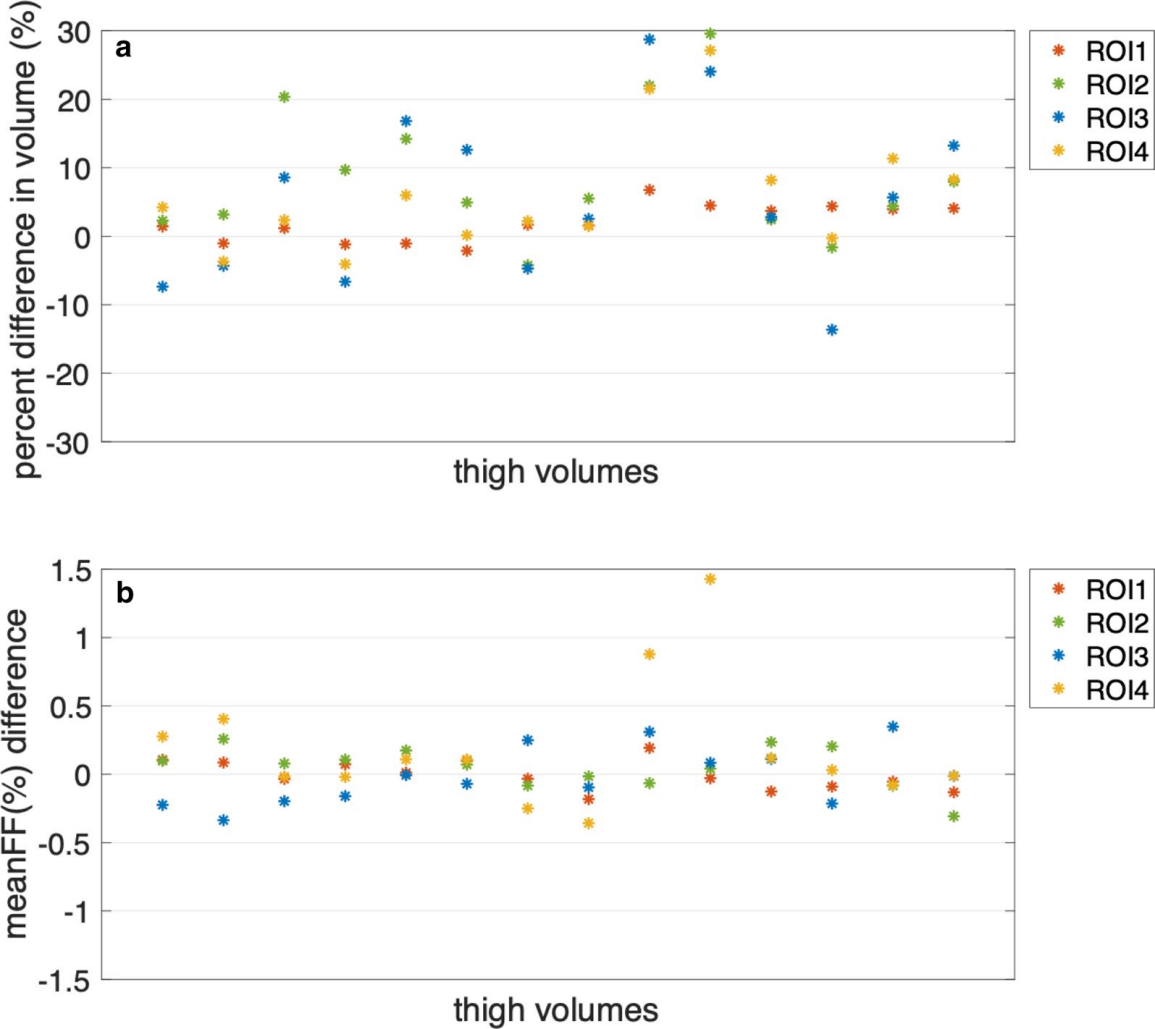
**a** Percent differences in volume between manual and automated segmentation for the 26 thigh volumes in the testing set. The average of the absolute differences was 7.57%. **b** The differences in meanFF between manual and automated segmentation for the 26 thigh volumes in the testing set. The average of the absolute differences was 0.17%

Figure 5 demonstrates the percent differences in volume and meanFF differences between manual and automated segmentation for each thigh volume in the testing set (the values of each thigh volume with repeated scans were averaged). The average percent difference in volume (absolute values) was 7.57%. As ROI2 and ROI3 are relatively small muscle regions, the percent difference in volume could be large even if the volume difference was small. The differences in meanFF of all thigh volumes were within an acceptable range (− 0.5 to 1.5%). The average of the absolute differences in meanFF was 0.17%.

Table 2 demonstrates the ICCs of meanFF in the four ROIs based on the repeated scans. As shown, CNN-based segmentation method produced more reproducible fat fraction measures than manual segmentation in all ROIs.

**Table 2:** Reproducibility analyses calculated by ICCs on meanFF of the repeated scans

ICCs [95% confidence interval]	Manual segmentation	CNN-based automated segmentation
ROI1	0.889 [0.645,0.982]	0.910 [0.702,0.985]
ROI2	0.604 [0.126,0.922]	0.639 [0.171,0.930]
ROI3	0.889 [0.645,0.982]	0.913 [0.710,0.986]
ROI4	0.942 [0.798,0.991]	0.968 [0.884,0.995]
Overall	0.902 [0.820,0.953]	0.921 [0.852,0.962]

ROI1, quadriceps femoris; ROI2, sartorius; ROI3, gracilis; ROI4, hamstrings

### Quantitative analysis results

**Fig. 6:**
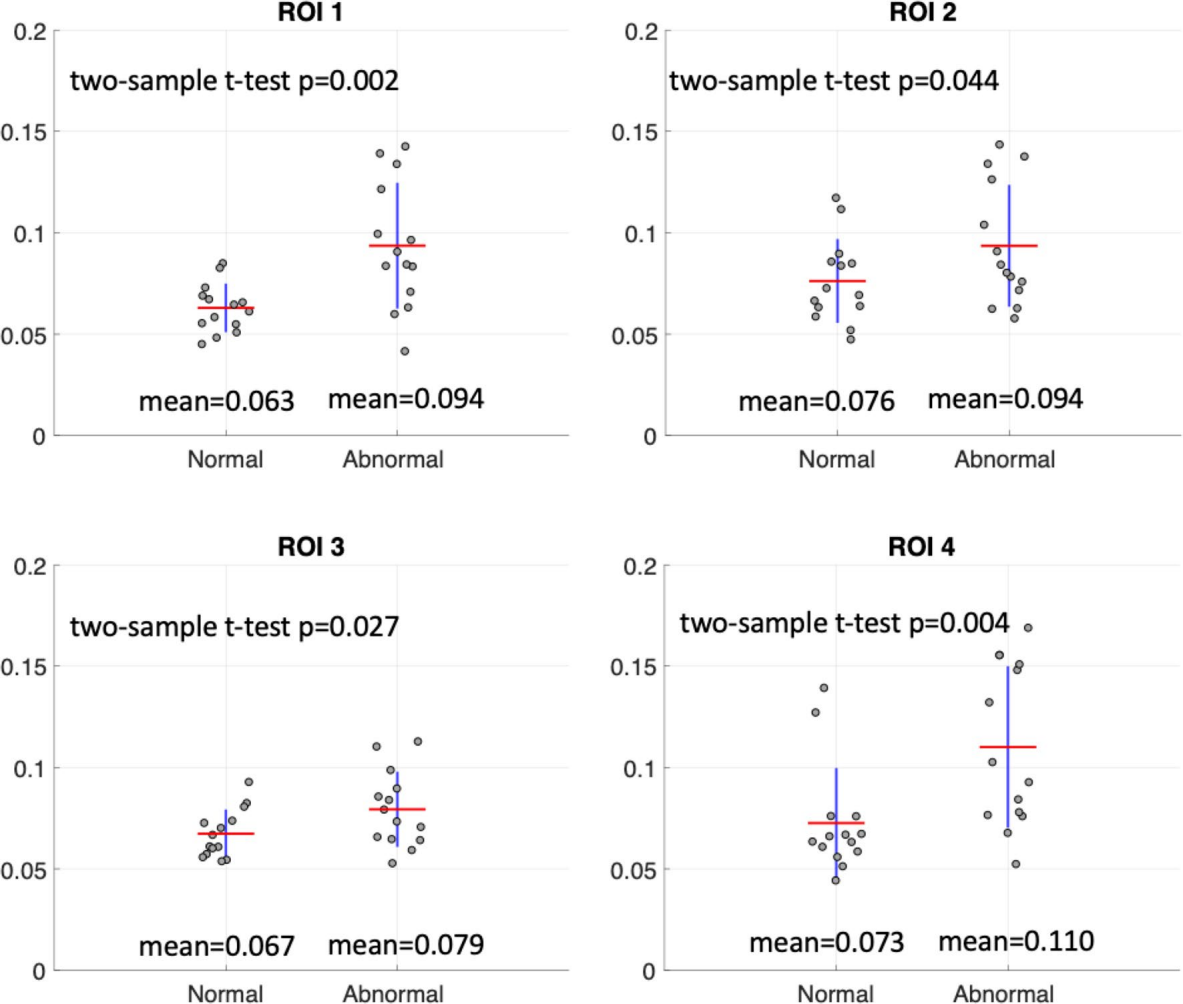
A quantitative analysis on meanFF using the CNN segmentation based on 14 normal and 14 abnormal thigh volumes of the clinical data. The averages of meanFF values (indicated by the red lines) and the two-sample *t* test *p* values are shown in each plot

Figure 6 illustrates the meanFF differences between the 14 normal and 14 abnormal thigh volumes in Dataset 2 calculated using the CNN-based automated ROIs. For all ROIs, abnormal thighs showed significantly higher fat fraction than normal thighs, although a clear threshold could not be defined based on this preliminary analysis.

## Discussion

In this work, we adopt the U-net architecture to train a CNN using fat–water decomposition MRI for thigh muscle segmentation. Time-efficient thigh muscle segmentation is a major challenge in moving from a primarily qualitative assessment of thigh muscle MRI in clinical practice, to potentially more accurate and quantitative methods. Compared to the time-consuming and labor-intensive manual annotation, which ordinarily takes at least 5–6 h, the proposed fully automated method provided sufficiently accurate segmentation taking between 10–30 s for each thigh volume. In terms of fat fraction quantification, the automated segmentation demonstrated higher reproducibility in fat fraction estimation compared to manual segmentation (overall ICCs: 0.921 vs. 0.902). A reproducible measurement allows serial monitoring, which can be useful, particularly if there is an intervention to allow for the detection of disease progression or for evaluation of treatment efficacy. Furthermore, the automated segmentation enabled the quantitative evaluation of fat infiltration in thigh muscles and showed potential for assisting diagnosis or surveillance when applied to clinical data.

This CNN segmentation method was trained using images from two datasets that were acquired by research used six-point Dixon (Dataset 1) and also clinically implemented three-point Dixon (Dataset 2). Although the two datasets were obtained with different sequences and scanners, our generalized network demonstrates satisfying segmentation accuracy in both datasets. This an important strength of our segmentation network as it can be applied to both clinical and research data for further studies. Both three-point and the more advanced six-point Dixon methods have been widely used for quantifying muscle fat content [2]. The six-point Dixon method used in Dataset 1 [20] not only addresses magnetic field inhomogeneities but also corrects for confounding factors including T1 bias, T2* decay and multispectral complexity of fat protons, in order to perform more accurate fat–water separation and obtain the PDFF maps. PDFF is a standardized biomarker of tissue fat concentration that reflects the fundamental property of tissue [14]. If available, such a method is preferred for more accurate muscle fat quantification, as suggested by previous studies [2, 15]. For three-point Dixon method, although some confounding factors exist, it has also been widely used to measure the fat replacement of skeletal muscle [2] due to its higher availability in clinical practice, and its inter- and intra-rater reliability has also been validated [5, 15, 16].

This automated, fast and reproducible thigh muscle segmentation is expected to be useful for quantitative assessment of the intramuscular fat infiltration in clinical practice. We thereby performed a preliminary analysis using our local clinical data, which demonstrates the feasibility of using this CNN-based thigh muscle segmentation method in differentiating the normal thighs and the abnormal thighs with fat infiltration. Significant differences between normal and abnormal thighs were detected in all four ROIs using the CNN-based automated segmentation.

Our method shows advantages compared to other studies that tackled this challenging task in recent years [21–25]. Kemnitz et al. [21] developed a semiautomated thigh muscle segmentation method using an active shape model. In order to achieve high agreement with manual segmentation, this method required manual interaction to modify the individual thigh component masks as the first step before applying the segmentation. Later in 2019, they also applied a U-net deep learning architecture for fully automated thigh muscle segmentation [22]. The segmentation was performed only for a particular anatomical location (33% of the femoral bone), which might not be used for volumetric analysis. In addition, these two studies used nonquantitative T1-weighted MRI, which limited their applications in quantitative studies. Rodrigues et al. [23] developed an automated thigh muscle segmentation method based on local texture analysis using three-point Dixon MRI data. They segmented the entire muscle region without distinguishing any muscle groups. Mesbah et al. [24] segmented three thigh muscle groups on the fat and water images utilizing a 3-D Joint Markov Gibbs Random Field model. The approach was performed on the preselected 50 central slices in a total of seven steps, which might make it difficult to apply in clinical settings. Moreover, the above studies did not perform reproducibility analysis. Ogier et al. [25] designed a semiautomatic segmentation pipeline of individual leg muscles using two-point Dixon and three-point Dixon images based on automatic propagation through nonlinear registrations of initial delineation. The reproducibility was also assessed on four subjects scanned twice on the same day. However, the initial manual thigh muscle segmentation of three slices was required. It is also noted that the two-point Dixon technique can suffer from phase errors due to static field inhomogeneity, which should be used with caution in quantitative measurement [34]. Our CNN-based automated method for thigh muscle segmentation on the whole volumes is designed for quantitative fat–water decomposition MRI (three-point and six-point Dixon sequences), which could be further used to identify potential image-based biomarkers. It produces sufficiently accurate segmentation of four muscle groups in seconds and exhibits high reproducibility compared with manual segmentation.

The current study has some limitations. First, the number of subjects was relatively small. In order to reduce the effect of the small dataset, the sample size was doubled after separating the left and right thigh volumes, and our network was trained in 2D using a total of 4968 slices. However, more data are needed to further improve the segmentation performance and also validate its reproducibility. Second, the current CNN segmentation was not suitable for patients with markedly severe fat infiltration (i.e., whole muscle group involvement obscuring the muscle boundary), as we had limited data of such cases to train the network. Third, for the preliminary quantitative analysis, the pathology results, usually used as a gold standard, were missing for our dataset. In addition, only meanFF was calculated, and no clear thresholds were found to distinguish the normal and abnormal thighs in our preliminary results. Fourth, it is noted that both segmentations yielded only moderate reproducibility in ROI2, which suggests ROI2, the sartorius muscle, might not be suitable for quantitative determination of fat infiltration. This is probably because ROI2 is the smallest muscle region among these four ROIs. For a very small ROI, a little difference in ROI delineation can lead to a large difference in fat fraction estimation. Finally, the segmentation network was trained and tested only based on these two datasets. Although satisfying results were achieved for these two datasets, external validation using an independent dataset acquired with a different scanner is further needed to ensure the reproducibility and feasibility of this segmentation method in clinical practice. Further studies are also needed to establish age-specific reference ranges as well as to improve and validate the segmentation performance across the spectrum of diseases. In addition, more image-based features or biomarkers can be investigated in future studies with a larger dataset, and other factors, including age, sex and body mass index, need to be considered.

## Conclusions

In conclusion, we present a deep learning-based thigh muscle segmentation method using fat–water decomposition MRI. This fast and automated method exhibits excellent segmentation accuracy and higher reproducibility in fat fraction estimation compared to manual segmentation, which would be beneficial to clinical practice such as quantifying fat infiltration in thigh muscles associated with ageing, or in conditions such as neuromuscular disorders. Moreover, reproducible segmentation allows serial monitoring which can be particularly useful for detection of disease progression or for evaluation of treatment efficacy.

## Supplementary information

**Additional file 1: Table S1.** Subjects’ demographic and clinical characteristics.

## Data Availability

The datasets used and/or analyzed during the current study are available from the corresponding author on reasonable request.

## Abbreviations

CNN: Convolutional neural network
FF: Fat fraction
GRE: Gradient echo
ICC: Intraclass correlation coefficient
PDFF: Proton density fat fraction
ROI: Region-of-interest
TE: Echo time
TR: Repetition time
