# Supplementary material for: Deep learning-based thigh muscle segmentation for reproducible fat fraction quantification using fat–water decomposition MRI
Source: Insights Imaging. 2020 Nov 30;11:128. doi: 10.1186/s13244-020-00946-8 (PMC7704819; doi:10.1186/s13244-020-00946-8)
Supplement: Supplementary file 1 — Additional file 1: Table S1. Subjects’ demographic and clinical characteristics. [file 13244_2020_946_MOESM1_ESM.docx]

**Electronic Supplemental Material**

**Supplementary Table S1.** Subjects’ demographic and clinical characteristics

| Datasets | Dataset-1: reference database MyoSegmenTUM | | Dataset-2: local clinical data |
| --- | --- | --- | --- |
| Number of Subjects | 19 | | 21 |
| Sex | male/female=14:5 | | male/female=14:7 |
| Age (years)  (mean ± SD) | healthy volunteers | 29.1 ± 7.7 | 56.4 ± 14.5 |
|  | patients | 52.8 ± 8.9 |  |
| Characteristics | Healthy volunteers (n=15)  Patients with different neuromuscular diseases:  myotonic dystrophy type 2 (n = 2); limb girdle muscular dystrophy 2A (n = 1); amyotrophic lateral sclerosis (n = 1). | | Myositis:  drug-induced anti-HMGCR (n=2); other causes myositis, or otherwise unspecified (n=3).  Myopathy:  anti-SRP-associated NAM (n=1); PMA progressive muscular atrophy (n=1); muscular dystrophy FSHD type1 (n=1); X-linked recessive Kennedy's disease (n=1); biopsy-confirmed non-specific myopathy (n=2); clinically myopathy pending confirmation (n=2).  Clinically normal:  Radiologically normal but no biopsy (n=7);  Radiologically normal with biopsy (n=1). |
